# {4,6-Bis[(*E*)-1-methyl-2-(pyridin-2-yl­methyl­idene)hydrazinyl]pyrimidine-κ^3^ *N*,*N*′,*N*′′}dichloridomanganese(II)

**DOI:** 10.1107/S1600536811043352

**Published:** 2011-11-05

**Authors:** Bartosz Marzec, Mariyatra Mahimaidoss, Lei Zhang, Thomas McCabe, Wolfgang Schmitt

**Affiliations:** a School of Chemistry and CRANN, Trinity College, University of Dublin, College Green, Dublin 2, Republic of Ireland

## Abstract

In the title compound, [MnCl_2_(C_18_H_18_N_8_)], the geometry around the Mn^II^ centre is distorted square-pyramidal. In the crystal structure, mol­ecules pack *via* weak C—H⋯N and C—H⋯Cl inter­actions.

## Related literature

For the synthesis of the ligand, see: Schmitt *et al.* (2003 ▶). For the coordination chemistry of similar ligand types, see: Stadler *et al.* (2005 ▶, 2006 ▶). For coordination chemistry of similar complexes that contain Mn—N bonds, see: Romain *et al.* (2011 ▶). For a related structure containing copper(II) ions, see: Marzec *et al.* (2011 ▶).
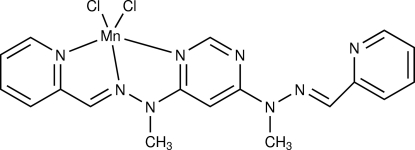


## Experimental

### 

#### Crystal data

[MnCl_2_(C_18_H_18_N_8_)]
*M* *_r_* = 472.24
Triclinic, 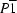

*a* = 8.8355 (12) Å
*b* = 10.0972 (14) Å
*c* = 12.1466 (17) Å
α = 72.571 (3)°
β = 77.694 (3)°
γ = 75.700 (3)°
*V* = 990.4 (2) Å^3^
*Z* = 2
Mo *K*α radiation
μ = 0.96 mm^−1^
*T* = 123 K
0.15 × 0.10 × 0.08 mm

#### Data collection

Bruker SMART CCD diffractometer
Absorption correction: multi-scan (*SADABS*; Blessing, 1995 ▶) *T*_min_ = 0.891, *T*_max_ = 0.926
13578 measured reflections
4889 independent reflections
4095 reflections with *I* > 2σ(*I*)
*R*_int_ = 0.029

#### Refinement

*R*[*F* ^2^ > 2σ(*F* ^2^)] = 0.039
*wR*(*F* ^2^) = 0.088
*S* = 1.04
4889 reflections
264 parameters
H-atom parameters constrained
Δρ_max_ = 0.47 e Å^−3^
Δρ_min_ = −0.24 e Å^−3^

### 

Data collection: *SMART* (Bruker, 2007 ▶); cell refinement: *SAINT* (Bruker, 2007 ▶); data reduction: *SAINT*; program(s) used to solve structure: *SHELXS97* (Sheldrick, 2008 ▶); program(s) used to refine structure: *SHELXL97* (Sheldrick, 2008 ▶); molecular graphics: *CrystalMaker* (Palmer, 2011 ▶) and *DIAMOND* (Brandenburg, 1998 ▶); software used to prepare material for publication: *SHELXL97*.

## Supplementary Material

Crystal structure: contains datablock(s) I, global. DOI: 10.1107/S1600536811043352/su2330sup1.cif

Structure factors: contains datablock(s) I. DOI: 10.1107/S1600536811043352/su2330Isup2.hkl

Supplementary material file. DOI: 10.1107/S1600536811043352/su2330Isup3.mol

Additional supplementary materials:  crystallographic information; 3D view; checkCIF report

## Figures and Tables

**Table 1:** Hydrogen-bond geometry (Å, °)

*D*—H⋯*A*	*D*—H	H⋯*A*	*D*⋯*A*	*D*—H⋯*A*
C2—H2⋯N3^i^	0.95	2.50	3.358 (3)	151
C18—H18⋯Cl3^ii^	0.95	2.80	3.508 (2)	133

Symmetry codes: (i) 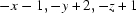; (ii) 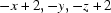.

**Fig. 1.**
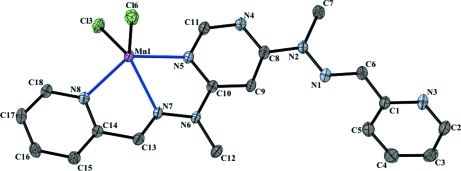
The molecular structure of the title complex, showing the numbering scheme and displacement ellipsoids drawn at 50% probability level (H atoms have been omitted for clarity).

**Fig. 2.**
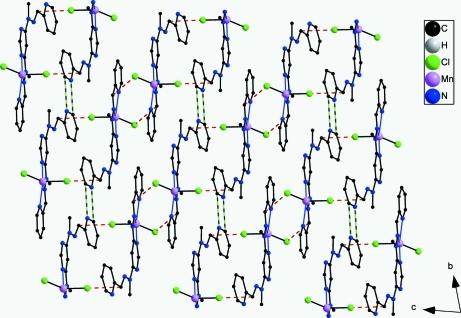
Crystal packing of the title complex, viewed along the a-axis, showing the C—H···Cl interactions as dashed red lines (H atoms have been omitted for clarity).

## supplementary crystallographic information

### Comment

(Pyridin-2-ylmethylene)hydrazinyl)pyrimidine-based ligands are an interesting class of compounds due to their functionalities and their geometrical features that might contribute significantly to the field of supramolecular chemistry. Lehn and coworkers recently reported that this class of compounds can systematically and modularly be extended resulting in N-functional ligand strands with varying dimensions (Stadler *et al.*, 2005, 2006; Schmitt *et al.*, 2003). The modular nature of the ligands enhances their appeal to be used in coordination chemistry. We present herein, the crystal structure of a manganese(II) complex of the above mentioned ligand.

In the title complex the manganese(II) atom is penta-coordinated by three N atoms of the organic ligand (4,6-bis[(*N*-methyl-2-(pyrindin-2-ylmethylene)hydrazinyl]pyrimidine) and two Cl atoms (Fig. 1). The coordination geometry of the central Mn^II^ ion can be best described as distorted square pyramidal. The N5, N7, N8 and Cl3 atoms form the basal plane and the Cl6 Cl atom occupys the apical position. The bond distances between the N atoms and the metal ion vary between 2.2227 (16) Å [Mn1—N5] and 2.2628 (16) Å [Mn1—N7]. The Mn—Cl bond distances are 2.3695 (6) Å for Mn1—Cl3 and 2.3453 (7) Å for Mn1—Cl6. The angle between the central metal ion and the Cl atoms [Cl3—Mn1—Cl6] is equal to 113.06 (2)°. The angles between the Mn^II^ ion and the coordinating atoms located in the basal plane vary between 69.14 (6)° [N5—Mn1—N7] and 103.08 (4)° [Cl3—Mn1—N8]. The configuration around atoms C6 and C13 is assigned to be *E*, as the torsion angles N2—N1—C6—C1 and N6—N7—C13—C14 are 176.36 (17)° and -177.09 (16)°, respectively.

In the crystal the complex molecules are connected by intermolecular C—H···Cl and C—H···N hydrogen bonds (Fig. 2). The former exists between the non-coordinating N atom N3 and C atom C2, and the latter between the Cl atom Cl3 and C atom C18, see Table 1 for details.

A related structure that contains copper(II) ions was reported on by us recently (Marzec *et al.*, 2011).

### Experimental

4,6-Bis[*N*-methyl-2-(pyrindin-2-ylmethylene)hydrazinyl]pyrimidine (0.007 g, 0.025 mmol) was dissolved in 5 ml of dichloromethane and 4.50 ml of methanol. Then 0.50 ml of a methanolic 0.1 *M* manganese(II)chloride tetrahydrate solution was added and the mixture was left for slow evaporation. Orange, block-shaped crystals of the title compound were collected after 4 d. Yield: *ca* 85%.

### Refinement

The H atoms were positioned geometrically and were included in the refinement in a riding model approximation: C—H = 0.95 and 0.98 Å for CH and CH_3_ H atoms, respectively, with *U*_iso_(H) = kU_eq_(C), where k = 1.5 for CH_3_ H atoms, and k = 1.2 for all other H atoms.

### Crystal data

**Table:**

[MnCl_2_(C_18_H_18_N_8_)]	*Z* = 2
*M**_r_* = 472.24	*F*(000) = 482
Triclinic, *P*1	*D*_x_ = 1.583 Mg m^−^^3^
Hall symbol: -P 1	Mo *K*α radiation, λ = 0.71073 Å
*a* = 8.8355 (12) Å	Cell parameters from 209 reflections
*b* = 10.0972 (14) Å	θ = 1.8–28.4°
*c* = 12.1466 (17) Å	µ = 0.96 mm^−^^1^
α = 72.571 (3)°	*T* = 123 K
β = 77.694 (3)°	Block, orange
γ = 75.700 (3)°	0.15 × 0.10 × 0.08 mm
*V* = 990.4 (2) Å^3^	

### Data collection

**Table:**

Bruker SMART CCD diffractometer	4889 independent reflections
Radiation source: fine-focus sealed tube	4095 reflections with *I* > 2σ(*I*)
graphite	*R*_int_ = 0.029
ω and φ scans	θ_max_ = 28.4°, θ_min_ = 1.8°
Absorption correction: multi-scan (*SADABS*; Blessing, 1995)	*h* = −11→11
*T*_min_ = 0.891, *T*_max_ = 0.926	*k* = −13→13
13578 measured reflections	*l* = −16→16

### Refinement

**Table:**

Refinement on *F*^2^	Primary atom site location: structure-invariant direct methods
Least-squares matrix: full	Secondary atom site location: difference Fourier map
*R*[*F*^2^ > 2σ(*F*^2^)] = 0.039	Hydrogen site location: inferred from neighbouring sites
*wR*(*F*^2^) = 0.088	H-atom parameters constrained
*S* = 1.04	*w* = 1/[σ^2^(*F*_o_^2^) + (0.0408*P*)^2^ + 0.5107*P*] where *P* = (*F*_o_^2^ + 2*F*_c_^2^)/3
4889 reflections	(Δ/σ)_max_ = 0.001
264 parameters	Δρ_max_ = 0.47 e Å^−^^3^
0 restraints	Δρ_min_ = −0.24 e Å^−^^3^

### Special details

**Table:**

Geometry. All e.s.d.'s (except the e.s.d. in the dihedral angle between two l.s. planes) are estimated using the full covariance matrix. The cell e.s.d.'s are taken into account individually in the estimation of e.s.d.'s in distances, angles and torsion angles; correlations between e.s.d.'s in cell parameters are only used when they are defined by crystal symmetry. An approximate (isotropic) treatment of cell e.s.d.'s is used for estimating e.s.d.'s involving l.s. planes.
Refinement. Refinement of *F*^2^ against ALL reflections. The weighted *R*-factor *wR* and goodness of fit *S* are based on *F*^2^, conventional *R*-factors *R* are based on *F*, with *F* set to zero for negative *F*^2^. The threshold expression of *F*^2^ > σ(*F*^2^) is used only for calculating *R*-factors(gt) *etc*. and is not relevant to the choice of reflections for refinement. *R*-factors based on *F*^2^ are statistically about twice as large as those based on *F*, and *R*-factors based on ALL data will be even larger.

### Fractional atomic coordinates and isotropic or equivalent isotropic displacement parameters (Å^2^)

**Table:**

	*x*	*y*	*z*	*U*_iso_*/*U*_eq_	
Mn1	0.67993 (3)	0.16794 (3)	0.82771 (3)	0.01792 (9)	
Cl3	0.79147 (6)	0.23004 (5)	0.96172 (5)	0.02639 (12)	
Cl6	0.83681 (6)	0.18893 (6)	0.64375 (4)	0.02952 (13)	
N1	0.01708 (19)	0.67410 (17)	0.66222 (14)	0.0208 (3)	
N2	0.14098 (19)	0.69997 (17)	0.69778 (15)	0.0212 (3)	
N3	−0.3287 (2)	0.85135 (18)	0.53910 (16)	0.0256 (4)	
N4	0.37974 (19)	0.59915 (17)	0.76727 (15)	0.0213 (3)	
N5	0.47733 (18)	0.34990 (17)	0.80430 (14)	0.0191 (3)	
N6	0.32711 (18)	0.19442 (17)	0.79741 (15)	0.0201 (3)	
N7	0.45792 (18)	0.09592 (16)	0.82524 (14)	0.0183 (3)	
N8	0.72286 (18)	−0.07073 (17)	0.88859 (14)	0.0193 (3)	
C1	−0.2119 (2)	0.7419 (2)	0.57423 (17)	0.0213 (4)	
C2	−0.4426 (2)	0.8238 (2)	0.49708 (19)	0.0282 (5)	
H2	−0.5268	0.8997	0.4731	0.034*	
C3	−0.4455 (3)	0.6926 (2)	0.48639 (19)	0.0291 (5)	
H3	−0.5288	0.6789	0.4553	0.035*	
C4	−0.3236 (3)	0.5808 (2)	0.52218 (19)	0.0294 (5)	
H4	−0.3217	0.4888	0.5160	0.035*	
C5	−0.2060 (2)	0.6057 (2)	0.56669 (18)	0.0257 (4)	
H5	−0.1215	0.5309	0.5921	0.031*	
C6	−0.0873 (2)	0.7771 (2)	0.61700 (17)	0.0219 (4)	
H6	−0.0844	0.8724	0.6113	0.026*	
C7	0.1690 (2)	0.8430 (2)	0.6747 (2)	0.0269 (4)	
H7A	0.0817	0.8981	0.7172	0.040*	
H7B	0.1756	0.8885	0.5908	0.040*	
H7C	0.2683	0.8385	0.7006	0.040*	
C8	0.2476 (2)	0.5803 (2)	0.74022 (16)	0.0192 (4)	
C9	0.2191 (2)	0.4463 (2)	0.75266 (17)	0.0202 (4)	
H9	0.1217	0.4339	0.7398	0.024*	
C10	0.3397 (2)	0.3324 (2)	0.78463 (16)	0.0178 (4)	
C11	0.4861 (2)	0.4824 (2)	0.79677 (18)	0.0218 (4)	
H11	0.5805	0.4943	0.8149	0.026*	
C12	0.1896 (2)	0.1539 (2)	0.7783 (2)	0.0257 (4)	
H12A	0.1624	0.0739	0.8428	0.039*	
H12B	0.2136	0.1263	0.7047	0.039*	
H12C	0.1001	0.2341	0.7744	0.039*	
C13	0.4659 (2)	−0.0363 (2)	0.83818 (17)	0.0211 (4)	
H13	0.3820	−0.0705	0.8253	0.025*	
C14	0.6114 (2)	−0.1315 (2)	0.87384 (16)	0.0189 (4)	
C15	0.6297 (2)	−0.2762 (2)	0.89248 (18)	0.0236 (4)	
H15	0.5485	−0.3155	0.8816	0.028*	
C16	0.7687 (2)	−0.3630 (2)	0.92733 (18)	0.0252 (4)	
H16	0.7850	−0.4627	0.9400	0.030*	
C17	0.8830 (2)	−0.3016 (2)	0.94323 (18)	0.0243 (4)	
H17	0.9791	−0.3585	0.9675	0.029*	
C18	0.8557 (2)	−0.1558 (2)	0.92326 (17)	0.0217 (4)	
H18	0.9348	−0.1145	0.9347	0.026*	

### Atomic displacement parameters (Å^2^)

**Table:**

	*U*^11^	*U*^22^	*U*^33^	*U*^12^	*U*^13^	*U*^23^
Mn1	0.01333 (14)	0.01712 (15)	0.02342 (16)	−0.00139 (10)	−0.00637 (11)	−0.00423 (11)
Cl3	0.0216 (2)	0.0294 (3)	0.0326 (3)	−0.00431 (19)	−0.0116 (2)	−0.0098 (2)
Cl6	0.0247 (3)	0.0315 (3)	0.0265 (3)	−0.0013 (2)	−0.0011 (2)	−0.0043 (2)
N1	0.0168 (8)	0.0219 (8)	0.0227 (8)	−0.0002 (6)	−0.0064 (6)	−0.0049 (7)
N2	0.0178 (8)	0.0173 (8)	0.0283 (9)	0.0002 (6)	−0.0094 (7)	−0.0047 (7)
N3	0.0191 (8)	0.0237 (9)	0.0309 (9)	0.0026 (7)	−0.0094 (7)	−0.0042 (7)
N4	0.0189 (8)	0.0192 (8)	0.0273 (9)	−0.0003 (6)	−0.0082 (7)	−0.0075 (7)
N5	0.0149 (7)	0.0200 (8)	0.0234 (8)	−0.0008 (6)	−0.0065 (6)	−0.0068 (6)
N6	0.0130 (7)	0.0175 (8)	0.0294 (9)	−0.0005 (6)	−0.0083 (6)	−0.0041 (7)
N7	0.0135 (7)	0.0185 (8)	0.0211 (8)	0.0012 (6)	−0.0057 (6)	−0.0039 (6)
N8	0.0175 (8)	0.0189 (8)	0.0213 (8)	−0.0021 (6)	−0.0062 (6)	−0.0038 (6)
C1	0.0180 (9)	0.0223 (9)	0.0205 (9)	0.0003 (7)	−0.0052 (7)	−0.0030 (8)
C2	0.0185 (10)	0.0324 (11)	0.0302 (11)	−0.0001 (8)	−0.0087 (8)	−0.0036 (9)
C3	0.0233 (10)	0.0387 (12)	0.0266 (11)	−0.0096 (9)	−0.0074 (8)	−0.0050 (9)
C4	0.0326 (12)	0.0275 (11)	0.0287 (11)	−0.0073 (9)	−0.0070 (9)	−0.0055 (9)
C5	0.0245 (10)	0.0232 (10)	0.0265 (10)	0.0001 (8)	−0.0075 (8)	−0.0034 (8)
C6	0.0199 (9)	0.0190 (9)	0.0244 (10)	0.0000 (7)	−0.0051 (8)	−0.0039 (8)
C7	0.0236 (10)	0.0183 (9)	0.0391 (12)	−0.0005 (8)	−0.0115 (9)	−0.0061 (9)
C8	0.0170 (9)	0.0216 (9)	0.0174 (9)	−0.0001 (7)	−0.0034 (7)	−0.0052 (7)
C9	0.0143 (9)	0.0209 (9)	0.0247 (10)	0.0001 (7)	−0.0073 (7)	−0.0048 (8)
C10	0.0153 (8)	0.0193 (9)	0.0191 (9)	−0.0020 (7)	−0.0034 (7)	−0.0060 (7)
C11	0.0175 (9)	0.0225 (10)	0.0275 (10)	−0.0017 (7)	−0.0082 (8)	−0.0079 (8)
C12	0.0159 (9)	0.0231 (10)	0.0401 (12)	−0.0034 (7)	−0.0101 (8)	−0.0075 (9)
C13	0.0176 (9)	0.0210 (9)	0.0254 (10)	−0.0031 (7)	−0.0071 (8)	−0.0048 (8)
C14	0.0164 (9)	0.0189 (9)	0.0202 (9)	−0.0023 (7)	−0.0041 (7)	−0.0036 (7)
C15	0.0225 (10)	0.0205 (9)	0.0293 (11)	−0.0035 (8)	−0.0082 (8)	−0.0064 (8)
C16	0.0282 (10)	0.0168 (9)	0.0291 (11)	0.0008 (8)	−0.0076 (9)	−0.0055 (8)
C17	0.0209 (10)	0.0245 (10)	0.0242 (10)	0.0039 (8)	−0.0087 (8)	−0.0044 (8)
C18	0.0176 (9)	0.0236 (10)	0.0238 (10)	−0.0025 (7)	−0.0072 (8)	−0.0045 (8)

### Geometric parameters (Å, °)

**Table:**

Mn1—N5	2.2227 (16)	C3—H3	0.9500
Mn1—N8	2.2580 (16)	C4—C5	1.373 (3)
Mn1—N7	2.2628 (16)	C4—H4	0.9500
Mn1—Cl6	2.3453 (7)	C5—H5	0.9500
Mn1—Cl3	2.3695 (6)	C6—H6	0.9500
N1—C6	1.279 (2)	C7—H7A	0.9800
N1—N2	1.365 (2)	C7—H7B	0.9800
N2—C8	1.373 (2)	C7—H7C	0.9800
N2—C7	1.459 (3)	C8—C9	1.396 (3)
N3—C2	1.339 (3)	C9—C10	1.382 (2)
N3—C1	1.345 (2)	C9—H9	0.9500
N4—C11	1.320 (2)	C11—H11	0.9500
N4—C8	1.348 (2)	C12—H12A	0.9800
N5—C11	1.334 (2)	C12—H12B	0.9800
N5—C10	1.350 (2)	C12—H12C	0.9800
N6—N7	1.355 (2)	C13—C14	1.463 (3)
N6—C10	1.385 (2)	C13—H13	0.9500
N6—C12	1.457 (2)	C14—C15	1.384 (3)
N7—C13	1.282 (2)	C15—C16	1.387 (3)
N8—C18	1.338 (2)	C15—H15	0.9500
N8—C14	1.348 (2)	C16—C17	1.380 (3)
C1—C5	1.394 (3)	C16—H16	0.9500
C1—C6	1.468 (3)	C17—C18	1.385 (3)
C2—C3	1.376 (3)	C17—H17	0.9500
C2—H2	0.9500	C18—H18	0.9500
C3—C4	1.389 (3)		
			
N5—Mn1—N8	138.73 (6)	N1—C6—H6	121.4
N5—Mn1—N7	69.14 (6)	C1—C6—H6	121.4
N8—Mn1—N7	71.34 (6)	N2—C7—H7A	109.5
N5—Mn1—Cl6	105.58 (5)	N2—C7—H7B	109.5
N8—Mn1—Cl6	98.09 (4)	H7A—C7—H7B	109.5
N7—Mn1—Cl6	109.23 (4)	N2—C7—H7C	109.5
N5—Mn1—Cl3	98.11 (4)	H7A—C7—H7C	109.5
N8—Mn1—Cl3	103.08 (4)	H7B—C7—H7C	109.5
N7—Mn1—Cl3	137.70 (4)	N4—C8—N2	117.01 (17)
Cl6—Mn1—Cl3	113.06 (2)	N4—C8—C9	122.41 (17)
C6—N1—N2	120.08 (17)	N2—C8—C9	120.58 (17)
N1—N2—C8	114.05 (15)	C10—C9—C8	116.66 (17)
N1—N2—C7	121.98 (15)	C10—C9—H9	121.7
C8—N2—C7	123.26 (16)	C8—C9—H9	121.7
C2—N3—C1	116.94 (18)	N5—C10—C9	121.65 (17)
C11—N4—C8	115.07 (17)	N5—C10—N6	116.17 (16)
C11—N5—C10	115.84 (16)	C9—C10—N6	122.17 (17)
C11—N5—Mn1	123.94 (12)	N4—C11—N5	128.05 (18)
C10—N5—Mn1	119.88 (12)	N4—C11—H11	116.0
N7—N6—C10	114.64 (15)	N5—C11—H11	116.0
N7—N6—C12	120.81 (15)	N6—C12—H12A	109.5
C10—N6—C12	124.50 (15)	N6—C12—H12B	109.5
C13—N7—N6	122.20 (16)	H12A—C12—H12B	109.5
C13—N7—Mn1	118.24 (12)	N6—C12—H12C	109.5
N6—N7—Mn1	119.14 (12)	H12A—C12—H12C	109.5
C18—N8—C14	117.69 (16)	H12B—C12—H12C	109.5
C18—N8—Mn1	125.94 (13)	N7—C13—C14	116.34 (17)
C14—N8—Mn1	115.79 (12)	N7—C13—H13	121.8
N3—C1—C5	122.57 (18)	C14—C13—H13	121.8
N3—C1—C6	115.26 (17)	N8—C14—C15	122.81 (17)
C5—C1—C6	122.13 (17)	N8—C14—C13	116.59 (16)
N3—C2—C3	124.18 (19)	C15—C14—C13	120.59 (17)
N3—C2—H2	117.9	C14—C15—C16	118.86 (18)
C3—C2—H2	117.9	C14—C15—H15	120.6
C2—C3—C4	118.28 (19)	C16—C15—H15	120.6
C2—C3—H3	120.9	C17—C16—C15	118.62 (18)
C4—C3—H3	120.9	C17—C16—H16	120.7
C5—C4—C3	118.8 (2)	C15—C16—H16	120.7
C5—C4—H4	120.6	C16—C17—C18	119.14 (18)
C3—C4—H4	120.6	C16—C17—H17	120.4
C4—C5—C1	119.22 (19)	C18—C17—H17	120.4
C4—C5—H5	120.4	N8—C18—C17	122.87 (18)
C1—C5—H5	120.4	N8—C18—H18	118.6
N1—C6—C1	117.14 (18)	C17—C18—H18	118.6
			
C6—N1—N2—C8	−177.44 (18)	N3—C1—C6—N1	173.05 (18)
C6—N1—N2—C7	−6.8 (3)	C5—C1—C6—N1	−9.2 (3)
N8—Mn1—N5—C11	160.34 (14)	C11—N4—C8—N2	−173.62 (17)
N7—Mn1—N5—C11	177.93 (17)	C11—N4—C8—C9	5.6 (3)
Cl6—Mn1—N5—C11	−77.03 (16)	N1—N2—C8—N4	174.36 (16)
Cl3—Mn1—N5—C11	39.75 (16)	C7—N2—C8—N4	3.8 (3)
N8—Mn1—N5—C10	−26.59 (19)	N1—N2—C8—C9	−4.8 (3)
N7—Mn1—N5—C10	−9.01 (13)	C7—N2—C8—C9	−175.38 (19)
Cl6—Mn1—N5—C10	96.04 (14)	N4—C8—C9—C10	−5.5 (3)
Cl3—Mn1—N5—C10	−147.19 (14)	N2—C8—C9—C10	173.61 (17)
C10—N6—N7—C13	−178.82 (18)	C11—N5—C10—C9	3.3 (3)
C12—N6—N7—C13	−1.4 (3)	Mn1—N5—C10—C9	−170.32 (14)
C10—N6—N7—Mn1	−6.4 (2)	C11—N5—C10—N6	−177.21 (17)
C12—N6—N7—Mn1	171.03 (14)	Mn1—N5—C10—N6	9.2 (2)
N5—Mn1—N7—C13	−179.21 (16)	C8—C9—C10—N5	0.8 (3)
N8—Mn1—N7—C13	−11.35 (14)	C8—C9—C10—N6	−178.64 (17)
Cl6—Mn1—N7—C13	80.91 (15)	N7—N6—C10—N5	−1.6 (2)
Cl3—Mn1—N7—C13	−100.42 (15)	C12—N6—C10—N5	−178.96 (18)
N5—Mn1—N7—N6	8.07 (13)	N7—N6—C10—C9	177.86 (17)
N8—Mn1—N7—N6	175.93 (15)	C12—N6—C10—C9	0.5 (3)
Cl6—Mn1—N7—N6	−91.81 (13)	C8—N4—C11—N5	−0.9 (3)
Cl3—Mn1—N7—N6	86.86 (14)	C10—N5—C11—N4	−3.4 (3)
N5—Mn1—N8—C18	−161.13 (14)	Mn1—N5—C11—N4	169.90 (16)
N7—Mn1—N8—C18	−178.46 (17)	N6—N7—C13—C14	−177.09 (16)
Cl6—Mn1—N8—C18	73.90 (16)	Mn1—N7—C13—C14	10.4 (2)
Cl3—Mn1—N8—C18	−42.16 (16)	C18—N8—C14—C15	−0.3 (3)
N5—Mn1—N8—C14	27.89 (18)	Mn1—N8—C14—C15	171.45 (15)
N7—Mn1—N8—C14	10.56 (13)	C18—N8—C14—C13	178.80 (17)
Cl6—Mn1—N8—C14	−97.09 (13)	Mn1—N8—C14—C13	−9.4 (2)
Cl3—Mn1—N8—C14	146.86 (13)	N7—C13—C14—N8	−0.5 (3)
C2—N3—C1—C5	0.8 (3)	N7—C13—C14—C15	178.61 (19)
C2—N3—C1—C6	178.55 (18)	N8—C14—C15—C16	−0.3 (3)
C1—N3—C2—C3	−1.0 (3)	C13—C14—C15—C16	−179.37 (19)
N3—C2—C3—C4	0.6 (3)	C14—C15—C16—C17	0.6 (3)
C2—C3—C4—C5	0.1 (3)	C15—C16—C17—C18	−0.4 (3)
C3—C4—C5—C1	−0.2 (3)	C14—N8—C18—C17	0.6 (3)
N3—C1—C5—C4	−0.2 (3)	Mn1—N8—C18—C17	−170.26 (15)
C6—C1—C5—C4	−177.8 (2)	C16—C17—C18—N8	−0.2 (3)
N2—N1—C6—C1	176.38 (17)		

### Hydrogen-bond geometry (Å, °)

**Table:**

*D*—H···*A*	*D*—H	H···*A*	*D*···*A*	*D*—H···*A*
C2—H2···N3^i^	0.95	2.50	3.358 (3)	151
C18—H18···Cl3^ii^	0.95	2.80	3.508 (2)	133

Symmetry codes: (i) −*x*−1, −*y*+2, −*z*+1; (ii) −*x*+2, −*y*, −*z*+2.
